# Advances in Functional Cellulose Hydrogels as Electrolytes for Flexible Zinc-Ion Batteries

**DOI:** 10.3390/nano14201645

**Published:** 2024-10-13

**Authors:** Luo Xu, Yan Li, Jianxue Fu, Luwei Shi, Chunjie Li, Ruguang Ma

**Affiliations:** School of Materials Science and Engineering, Suzhou University of Science and Technology, Suzhou 215009, China

**Keywords:** cellulose, hydrogel, flexible Zinc-Ion Battery

## Abstract

Zinc-ion batteries (ZIBs) emerge as leading candidates for a flexible energy storage system, distinguished by high capacity, affordability, and inherent safety. The integration of hydrogel electrolytes, particularly those with saturated aqueous solvents, has significantly enhanced the electrochemical performance of ZIBs while preserving their essential flexibility. Nonetheless, challenges in electrochemical performance under specific conditions highlight the nascent stage of this technology, with numerous technical hurdles awaiting resolution. Addressing these challenges, recent investigations have leveraged the unique properties of cellulose hydrogel—namely, its exceptional toughness, tensile strength, extreme temperature resilience, stimulus responsiveness, and self-healing capabilities—to innovate multifunctional flexible zinc-based batteries. This paper conducts a comprehensive review of the physicochemical attributes of cellulose hydrogel electrolytes within ZIBs. We thoroughly analyze their performance under diverse environmental conditions, offering insights into the current landscape and their future potential. By examining these aspects, we aim to underscore the developmental prospects and the challenges that lie ahead for hydrogel electrolytes in ZIBs, paving the way for further advancement in this promising field.

## 1. Introduction

With the rapid development of science and technology, wearable devices with good flexibility, ranging from innovative displays to smart electronics and advanced implantable medical devices, are profoundly transforming our daily life [1,2]. The long-term operation of these devices hinges on the development of power sources, which are not only miniaturized and flexible but also exhibit robust security, lightweight, compact dimensions, and longevity. These systems must reliably operate under routine mechanical stresses, such as bending, folding, stretching, and even exposure to water, encountered in everyday utilization.

The prevalent lithium-ion batteries (LIBs), despite their widespread application as the power source in flexible devices, confront substantial challenges including the safety concerns arising from toxic and flammable organic electrolytes, constrained lithium resources, and elevated cost. These problems become particularly pronounced when LIBs are integrated into wearable or implantable devices that maintain direct contact with human body [3,4]. Furthermore, fabricating LIBs in the flexible formats while preventing electrolyte leakage and ensuring cleanliness for wearable applications proves to be a formidable task.

In this context, aqueous rechargeable batteries, particularly those based on potassium, magnesium, zinc, and aluminum, are gaining intensive attention as viable alternatives. Zinc-ion batteries (ZIBs) stand out as a superior replacement for LIBs, credited to their enhanced safety and rapid electrochemical reactions. The low redox potential of Zn (−0.76 V versus standard hydrogen electrode (SHE)), high theoretical specific capacity (820 mAh g^−1^), and global abundance underscore the cost-effectiveness of ZIBs [5,6]. Yet, the shift from traditional ZIBs employing liquid electrolytes to their flexible counterparts is pivotal in fulfilling the stringent requirements of next-generation wearable electronics, especially ensuring their resilience and functionality in a variety of real-world scenarios.

Within the architecture of flexible ZIBs, electrolyte is one of critical components, facilitating the connection between electrodes and serving as conduits for ion transport. Usually, (quasi)solid polymer is employed as electrolytes to integrate with zinc salts, notably those comprising a polymer matrix such as poly(ethylene oxide) [7]. This type of material was chosen for its resilience to mechanical stress, heat, and its capability to circumvent issues related to electrolyte evaporation and leakage [8,9]. However, these electrolytes exhibit a propensity to crystallize at ambient temperatures, a characteristic that severely restricts their ionic conductivity, impedes the mobility of Zn^2+^ ions, and confines the electrochemical stability window. These limitations collectively compromise the electrochemical performance of the batteries. Thus innovative solutions to enhance their efficacy are necessary [10].

Hydrogel electrolytes, recognized as a suitable material for flexible ZIBs, adeptly blend the high ionic conductivity of aqueous electrolytes with the structural integrity of solid polymer electrolytes. This unique combination overcomes the inherent limitations associated with both liquid and purely solid electrolytes by ensuring substantial water retention for efficient ion transport [11,12,13]. This advancement renders hydrogel electrolytes particularly suitable for application in flexible electronic devices. Historically, these versatile hydrogels were crafted from hydrophilic petroleum-derived polymers, such as polyacrylamide (PAM) and polyvinyl alcohol (PVA), prized for their chemical stability and mechanical strength but criticized for their lack of degradability and biological functionality [14]. In light of growing environmental concerns, recent research has been directed towards natural polymers—like cellulose, plant polyphenols, and chitosan—heralded for their non-toxic, degradable, biocompatible, and eco-friendly attributes, thereby marking a significant shift towards sustainable materials in the field of hydrogel electrolytes.

As cellulose stands as the most plentiful polysaccharide on Earth, found extensively in plants like cotton and produced by bacterial organisms, it represents a renewable resource for crafting hydrogel electrolytes, sustainably addressing the challenge of the energy storage. The burgeoning application of flexible ZIBs underscores the critical need for designing functionalized cellulose-based hydrogel electrolytes [15]. To maintain good mechanical robustness, researchers have ventured into diverse synthetic approaches to bestow these hydrogels with varied functional attributes suitable for flexible ZIBs. Thereby, this review delineates the cutting-edge in functionalized cellulose hydrogel electrolytes, exploring their crosslinking mechanisms, synthesis strategies, and functional applications including self-healing capabilities, enhanced ionic conductivity, broad operational temperature ranges, and the suppression of undesirable by-product formation. It also casts a forward-looking view on the prospective developments and research trajectories of cellulose hydrogel electrolyte materials, aiming to pave the way for the design and development in the future.

## 2. Fundamentals of Hydrogel and Cellulose

### 2.1. Hydrogels

**Figure 1:**
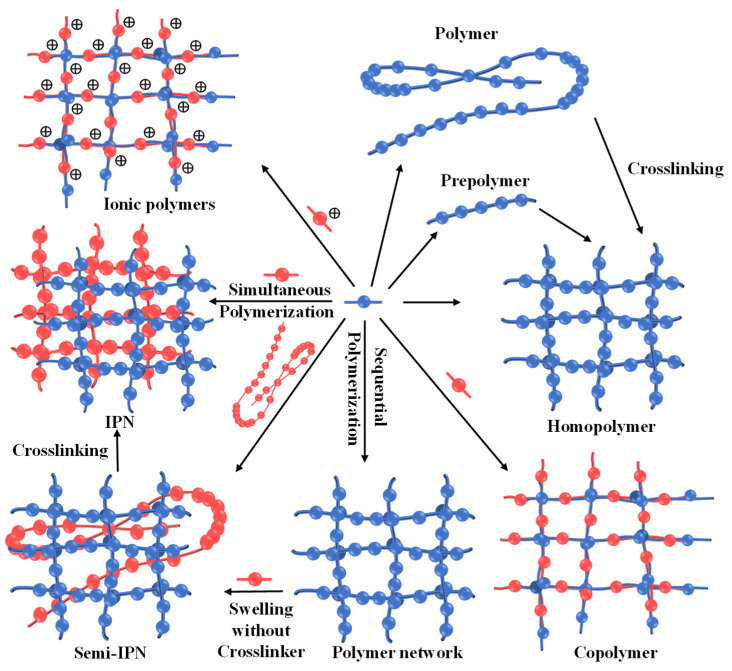
Scheme of different kinds of hydrogels and corresponding synthetic method. Reproduced with permission from Ref. [14]. Copyright© Elsevier 2023.

Hydrogels are a type of polymeric material that hold a large amount of water in their 3D networks, owing to their hydrophilic structure. The hydrophilic functional groups, such as hydroxyl groups (-OH), carbonyl groups (-C=O), carboxyl groups (-COOH), and amino groups (-NH_2_), help to store water and keep structural integrity by the weak physical hydrogen bond. Hydrogels have usually been described by researchers in different ways in the past decades. The popular definition is a crosslinked, water-swollen polymeric network formed by the crosslinking of monomers. In other words, it is a kind of polymeric substance that can swell and accommodate a certain amount of water inside the structure without dissolution. Hydrogels are generally synthesized by using three different types of polymers: homopolymers, copolymers, and interleaved polymer networks (IPNs) (Figure 1) [14]. Homopolymers only consist of one kind of monomer in the polymeric chains, while copolymers are comprised of two or more kinds of monomers. In contrast, there are usually two different types of polymer chains linked together in IPNs.

Hydrogel electrolytes, with their unique quasi-solid-state nature, effectively address the challenges of rechargeable aqueous ZIBs, including zinc dendrite formation leading to potential short circuits [16,17], side reactions such as hydrogen and oxygen evolution that compromise water content and battery capacity [18,19], and the dissolution of cathode materials in aqueous solutions. Evenly dispersing salt ions within the gel matrix enhances the ion conductivity, which is essential for electrochemical reactions [20,21]. Acting as separators, hydrogel electrolytes prevent short circuits and reduce the dissolution of active materials, when compared to liquid electrolytes. Good mechanical strength helps suppress the Zn dendritic growth at the anode [22]. Moreover, soft and viscous hydrogels can form intimate contact with the electrodes, thus decreasing the interface impedance and demonstrating good compatibility in flexible ZIBs.

Within the hydrated hydrogel matrix, the dissolved electrolytic ions are anchored by the charged functional groups present on the polymeric chains. This interaction not only localizes the ions but also contributes to the distinctive ionic conductivity of the hydrogel. Moreover, the elasticity of the crosslinked network accommodates significant water content and keeps the hydrogel matrix flexible. These features endow hydrogel materials with high ionic conductivity like in liquid while maintaining the structural stability characteristic of a quasi-solid state, rendering them exemplary for application in flexible batteries [23,24]. The conductivity of these hydrogels largely depends on the specific zinc salts, salt concentration, and overall water content within the hydrogel. At the conditions of high salt concentration and water saturation, diverse hydrogels exhibit varied characteristics and levels of ionic conductivity. This variability underscores the importance of selecting appropriate hydrogel formulations for energy storage applications, as the molecular structure, general properties, and ionic conductivity of hydrogels significantly impact their performance in flexible ZIBs. Some representative hydrogel materials used for fabricating electrolyte are summarized in Table 1.

The ideal hydrogel electrolytes for applications in flexible ZIBs must encapsulate a set of synergistic properties to ensure optimal performance. These properties include (a) high ionic conductivity to facilitate efficient charge transport, (b) robust mechanical strength coupled with intrinsic softness to withstand physical stress while maintaining flexibility, (c) excellent interfacial compatibility with electrodes to minimize resistance and enhance charge transfer, and (d) remarkable tolerance to extreme temperatures, ensuring stable operation under varying environmental conditions.

The strategic refinement of polymer chemistry through innovative approaches—such as the incorporation of additives, the grafting of functional groups onto the backbone of polymer, and the meticulous regulation of the structural parameters of hydrogel network—has yielded a plethora of novel hydrogel electrolytes. These advanced materials exhibit a wide array of functionalities that significantly bolster the performance metrics of ZIBs. Consequently, the resulting hydrogel electrolytes endow ZIBs with enhanced capacity, superior performance, sensitivity to external stimuli, and an extraordinary ability to withstand deformation. Such attributes are particularly advantageous for wearable technologies, where the adaptability of electrolyte to flexural stress without compromising battery performance is crucial. This burgeoning field of hydrogel electrolytes paves the way for versatile, high-capacity energy storage solutions, thereby expanding the frontier of flexible and wearable electronics [26,27].

### 2.2. Celluloses

**Figure 2:**
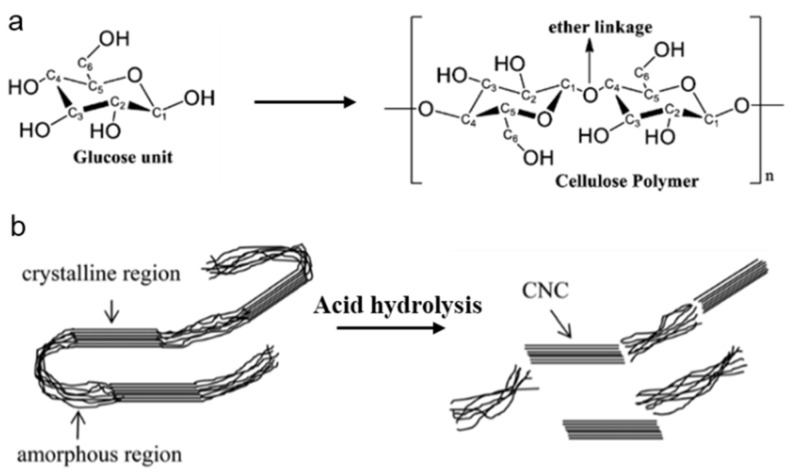
(**a**) Scheme of a glucose monomer unit and the corresponding cellulose polymer. Reproduced with permission from Ref. [34]. Copyright© The Royal Society of Chemistry, 2021. (**b**) Acid hydrolysis of semicrystalline cellulose fibers to synthesize CNCs. Reproduced with permission from Ref. [35]. Copyright© Wiley 2020.

Cellulose is a type of abundant natural biopolymer that provides unlimited raw materials for the ever-increasing demand of sustainable, non-toxic, and environmentally-friendly products. Additionally, cellulose is one of the main components of plant cell walls (e.g., cotton and woods), which is termed plant cellulose (PC) [28,29]. From the views of molecular level, cellulose is a linear, unbranched polymer linked by pyranose anhydrous glucose units at the C1 and C4 positions (Figure 2a) [30,31]. The degree of polymerization of cellulose (500–15,000) is highly dependent on the source of cellulose and physicochemical treatment methods. However, acidic and enzymatic (cellulase) hydrolysis can induce the decomposition of cellulose molecules, resulting in a significant reduction in molecular length and weight [32,33]. The -OH groups in cellulose molecules can form hydrogen bonds with the adjacent -OH groups, making cellulose crystallize. As a result, cellulose is a semicrystalline polymer. The amorphous regions of cellulose selectively get hydrolyzed upon acid hydrolysis, leading to the formation of pure cellulose nanocrystals (CNCs) (Figure 2b), which are usually used as reinforcing fillers in many industrial applications, such as composite manufacturing.

PC provides strength and structure to the plant, which is a rigid polymer consisting of hemicellulose and lignin (lignocellulose). Cellulose can also be synthetic polymers via some bacteria, i.e., acetobacter xylinum, which is called bacterial cellulose (BC). Glucose and sucrose are the main carbon sources for the synthesis of BC by ethyl xylate, but the yield of BC by glucose is higher [36]. Thanks to the absence of hemicellulose and lignin matrixes, BC is purer than PC, making the extraction and purification of cellulose easier. Actually, the chemical structure of BC is the same as that of PC, but BC demonstrates remarkably different physicochemical properties and degree of polymerization. For example, BC fibers usually have smaller diameter than PC fibers but better robustness and crystallinity than PC [36,37]. A high specific surface area of BC fibers and many -OH groups on the polymer chains enables BC to interact with H_2_O molecules well. This property makes BC suitable as adsorbents for wastewater treatment. Moreover, BC has also been widely applied in biomedical fields, pharmaceuticals, food, textile, and paper industries, owing to its unique molecular structure and properties [38].

The solubility of cellulose can be enhanced by destructing the ordered crystalline region, where a lot of hydrogen bonds exist between hydroxyl groups. Usually, there are three hydroxyl groups per repeating glucose unit. Hydroxyl groups in the cellulose backbone are subjected to etherification to form several cellulose derivatives, i.e., cellulose ethers, following Reaction (1) [32,33,39].

ROH + R*Cl → ROR* + HCl(1)

The modification of cellulose is usually conducted by two steps. Firstly, alkali cellulose is formed in sodium hydroxide solution (~15%), as shown in Reaction (2).

ROH + NaOH → RONa + H_2_O (2)

Then, side groups are incorporated into the cellulose backbone via an etherification process. Cellulose derivatives and the corresponding reaction mechanisms are shown in Table 2.

Cellulose ester derivatives (e.g., cellulose acetate, cellulose formate, and cellulose propionate, etc.) have also been successfully prepared by virtue of an esterification process. Cellulose acetate has been used in more areas than other esters. The solubility and physicochemical properties of cellulose ester derivatives are influenced by the side group substituents and degree of substitution (DS) substantially [36,40]. Cellulose derivatives usually have good biodegradability, biocompatibility, hydrophilicity, and structural tunability. So, they have been widely used in the biomedical science, pharmaceutical, agriculture, and food industries [36,41,42].

Over the past several years, the field of materials science has witnessed the emergence of advanced technologies and innovative synthesis methodologies, significantly contributing to the evolution of cellulose and its derivatives. These advancements have enhanced the intrinsic properties and functionalities of cellulose, thereby broadening its spectrum of applications across various domains.

The substantial body of literature, comprising numerous review articles and book chapters, underscores the extensive research and development efforts in understanding the sources, extraction processes, synthesis techniques, modifications, properties, and applications of cellulose. This is evidenced by the references [43,44,45,46,47,48], which collectively highlight the consensus in the scientific community on the importance of cellulose in modern materials science. For example, Seddiqi and colleagues have provided an in-depth analysis of the diverse sources and state-of-the-art formulation methods for cellulose and its derivatives, particularly emphasizing their suitability for biomedical applications [44]. Their discussion extends to an examination of cellulose in various forms, including fibers, microfibers, nanofibers, and micro and nanocrystals, elucidating their respective properties and application potential. Similarly, Liu et al. have focused on the utilization of cellulose and its derivatives in food packaging, presenting a critical review about manufacturing methods and functional properties of cellulose-based films [49]. Liu’s group has conducted a detailed exploration of cellulose applications in the oil field sector, investigating the applications of cellulose derivatives in the petroleum-related processes, such as drilling, cementing, and fracturing [50].

Moreover, the significance of cellulose and its derivatives extends to the synthesis of hydrogels [29,51,52,53,54,55,56]. This underlines the critical role of cellulose derivatives in advancing the frontiers of materials science, thereby offering innovative solutions to contemporary challenges across diverse sectors. In the realm of hydrogel construction, cellulose serves as a foundational element and is predominantly utilized in two distinct forms. The first form, nanocellulose, encompasses cellulose nanofibers and cellulose nanocrystals, which are derived through meticulous processes such as acid hydrolysis and high-pressure homogenization. These processes meticulously dismantle the macromolecular structure of cellulose, yielding nanoscale cellulose particles. Additionally, the bio-induced synthesis of bacterial nanocellulose, facilitated by specific strains of microbial bacteria, represents another prevalent method in the assembly of cellulose hydrogels. The second form pertains to cellulose solutions, which are homogeneous mixtures created by dissolving natural cellulose in specific solvents, including but not limited to alkali/urea systems and ionic liquids. The crux of constructing effective cellulose hydrogels lies in the intricate interactions between cellulose molecular chains within the gel matrix. By meticulously modulating the crosslinking mechanisms or incorporating heterogeneous components of varied functionalities, the structural and functional attributes of cellulose hydrogels can be finely modulated to meet specific requirements.

Cellulose-based hydrogels are usually fabricated either by employing native cellulose or by utilizing cellulose derivatives, which undergo crosslinking via chemical and/or physical methods. These approaches to hydrogel formation underscore cellulose’s versatility as raw materials, enabling the creation of hydrogels with customized properties for diverse applications. This adaptability, coupled with the renewable nature of cellulose, underscores its significance in advancing hydrogel technology, highlighting its potential to contribute to sustainable materials science and engineering solutions.

## 3. Crosslinking of Cellulose Hydrogels

**Figure 3:**
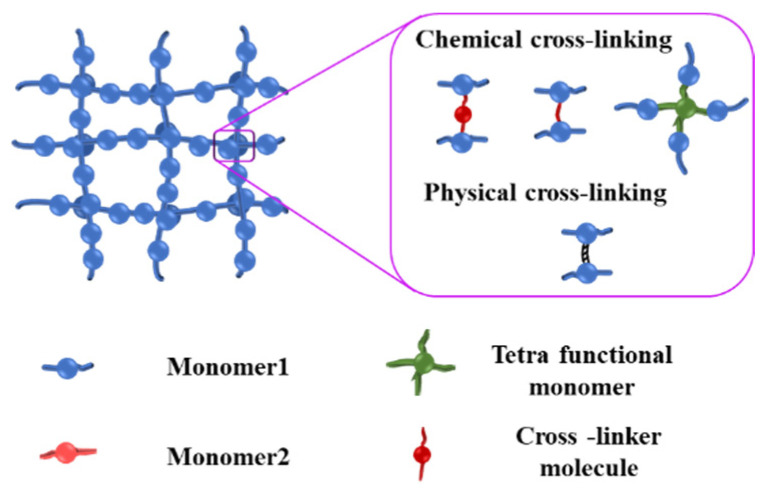
Scheme of physical crosslinking and chemical crosslinking. Reproduced with permission from Ref. [14]. Copyright© Elsevier 2023.

Crosslinking means the bonding or linking between two polymeric chains. Upon crosslinking, the movement of polymer chains is restricted and the polymer solution becomes a (quasi-)solid “gel”. After crosslinking, the as-formed polymers or hydrogels demonstrate larger molecular weight, higher mechanical strength, and better resistance to heat and solvents. Physical crosslinking and chemical crosslinking are mainly two crosslinking strategies employed in cellulose-based hydrogel synthesis (Figure 3). The properties of cellulose-based hydrogels, such as swelling, mechanical strength, elasticity, and release kinetics, strongly depend on the nature and degree of crosslinking. Radiation-assisted crosslinking has recently been developed to modulate the properties of hydrogels.

### 3.1. Physical Crosslinking

Physical crosslinking in cellulose hydrogels, predicated on non-covalent bonding mechanisms, fosters the formation of network junctions characterized by molecular entanglement and intermolecular forces, including hydrogen bonding, hydrophobic interactions, electrostatic interactions, and coordination bonds. This method eschews the creation of new chemical bonds at the polymer crosslinking points, thereby maintaining the integrity of the molecular structure of cellulose. The inherent reversibility of the physical crosslinking mechanism imbues the hydrogels with a dynamic 3D network structure. This attribute is particularly advantageous for engineering hydrogels that can exhibit high energy dissipation in response to external forces or possess self-healing capabilities, thus broadening their application spectrum.

For instance, Huang et al. have leveraged the ortho-phenol hydroxyl structures abundant in tannic acid (TA) as a molecular bridging agent to couple CNCs and PVA, resulting in the development of a nanocellulose-based physical hydrogel (TA-PVA/CNC) [57]. This hydrogel, characterized by its remarkable strength and toughness, relies on a network formed through multiple hydrogen bond crosslinking. Similarly, Mredha et al. [58] have fabricated high-strength, anisotropic hydrogels capable of self-repair by manipulating the arrangement of cellulose molecular chains via a method that alternates between drying and reswelling.

The strategy of physical crosslinking extends beyond singular physical interactions, permitting the incorporation of multiple types of physical forces within the same hydrogel system to endow the material with multifunctional capabilities. For example, Zhang et al. have synthesized a poly(acrylic acid-co-acrylamide) (PAAAM) hydrogel strengthened with quaternized tunicate CNCs (Q-TCNCs) and iron (Fe^3+^) through dual physical crosslinking mechanisms [59]. The Q-TCNCs serve a dual role as both an interface compatibility enhancer and a crosslinking agent, facilitating the formation of a network via electrostatic interactions between -N(CH_3_)^3+^ and -COO- groups. Simultaneously, a robust crosslinking network is established via the covalent bonds within PAAAM and ionic coordination between Fe^3+^ ions and -COO- groups of PAAAM. This intricate network structure showcases the potential of integrating various physical interactions to create cellulose hydrogels with enhanced mechanical properties and multifunctionality, including high strength and self-healing capabilities.

### 3.2. Chemical Crosslinking

Chemical initiation stands as a cornerstone technique in the realm of polymer synthesis, predominantly due to its efficacy in instigating polymerization processes. This method necessitates the incorporation of chemical initiators, which typically encompass compounds like ammonium cerium nitrate/nitric acid, ferrous sulfate/hydrogen peroxide, ammonium persulfate/sodium thiosulfate, potassium permanganate/sulfuric acid, and potassium persulfate. These initiators play a pivotal role in generating free radicals, thereby facilitating the polymerization of monomers into polymers.

For example, Suo et al. employed KPS and sodium metabisulfite as initiators to create a CMC-PAAAM hydrogel, which demonstrated exceptional absorbent capacities in distilled water and normal saline, reaching up to 920 g/g and 85 g/g, respectively [60]. This hydrogel exhibited notable water retention and salt tolerance properties. Furthermore, Yang et al. [61] synthesized a carboxymethyl cellulose-poly(acrylamide) hydrogel utilizing a REDOX system (ammonium persulfate/sodium sulfite), achieving a molecular weight of 7.5 × 10^6^ under optimal conditions. This hydrogel was characterized by enhanced viscosity, heat resistance, and salt resistance.

Despite these advancements, the chemical initiation process is not devoid of drawbacks, particularly concerning the generation of by-products that may lead to secondary pollution. This underscores the necessity for ongoing research and development efforts aimed at identifying and implementing novel triggering methods for polymer synthesis. Such innovations hold the potential to mitigate environmental impacts, representing a critical step forward in the pursuit of sustainable materials science. The quest for alternative initiation strategies that minimize or eliminate secondary pollutants is of paramount environmental importance, promising to pave the way for cleaner, greener polymer synthesis technologies.

### 3.3. Crosslinking Assisted by Radiation

**Figure 4:**
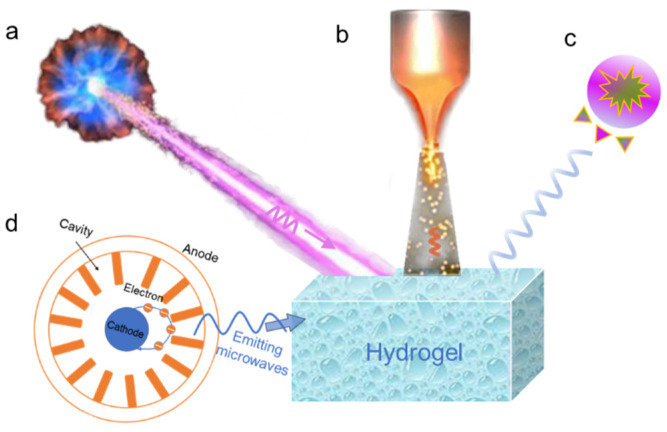
Radiation-assisted crosslinking of hydrogel, (**a**) gamma radiation, (**b**) electron-beam radiation, (**c**) ultraviolet radiation, and (**d**) microwave radiation.

Radiation interaction with materials has been recognized as an efficient strategy for constructing 3D polymer networks. This strategy offers many advantages over traditionally physical or chemical crosslinking methods, such as mild reaction conditions, negligible by-products, and quick gelation. As shown in Figure 4, radiation is generally divided into two categories: ionizing radiation and nonionizing radiation. Ionizing radiation can ionize the medium passing through via either direct or indirect interaction with the substance, including gamma radiation and electron beam. In contrast, nonionizing radiation cannot ionize material, due to a low ionizing potential, such as ultraviolet radiation and microwave radiation.

#### 3.3.1. Gamma Ray Radiation

Gamma ray radiation, characterized by its high-energy electromagnetic wave nature, plays a significant role in the field of polymer modification, despite the relatively low energy absorption rate by the medium. This necessitates prolonged irradiation times to effectuate significant modifications in polymers. In a notable application of this technology, Hiroki et al. embarked on the synthesis of mixed gels from a concentrated solution of CMC and carboxymethyl chitosan (CMCts) using γ-ray radiation [62]. Their research delved into the adsorption characteristics of the mixed gels towards Pb and Au ions by examining parameters such as gel fraction, swelling ratio, and gel strength. They found that the gel fraction escalated while the swelling ratio diminished with an increase in radiation dose. Notably, the mixed gels exhibited a remarkable adsorption capacity for Pb and Au ions, indicating the profound impact of the CMC and CMCts composition on the properties of mixed gels.

Similarly, Tamada et al. utilized ^60^Co γ-ray radiation to synthesize cellulose-based hydrogels modified with acrylonitrile and methacrylic acid [63]. Their research demonstrated that an optimal graft copolymerization outcome could be achieved with a 90% water solution of acrylonitrile and methacrylic acid, at a mass ratio of 4:1, under a nitrogen atmosphere. A total radiation dose of 200 kGy yielded the highest grafting rate. The resultant gel showcased an impressive absorption capacity for water, up to 12 times its own weight, when used to adsorb uranium-containing seawater. This marks it as a highly promising adsorbent for marine resource conservation.

Despite the simplicity and high grafting efficiency of radiation grafting reactions, they still have some drawbacks, including the potential for side reactions and the high cost associated with the process. These factors currently limit the scalability and broader application of radiation-induced polymer modifications. Therefore, further research is imperative to refine this method, reduce associated costs, and mitigate side reactions, thereby enhancing its viability for large-scale applications. This pursuit is crucial for advancing the development of environmentally sustainable and effective materials for widespread applications, such as pollution remediation and resource conservation.

#### 3.3.2. Electron-Beam Radiation

Electron-beam (e-beam) technology, facilitated by accelerators, offers a compelling alternative to γ-ray radiation for polymer modification. By producing beams that are both concentrated and uniformly directed, e-beam radiation achieves higher dose rates, making it markedly more energy-efficient, simpler to operate, and safer than its γ-ray counterpart. These attributes render e-beam radiation a superior method in specific applications, especially where precise control over radiation dose and direction is essential.

Ibrahim et al. [64] leveraged e-beam radiation to synthesize CMC/AAM hydrogels, meticulously examining the influence of radiation dose and polymer–monomer composition on the resultant gel strength. Their findings indicated that the most effective graft product was achieved at a radiation dose of 30 kGy, with an optimal CMC to AAM concentration ratio of 90/10. This composition significantly enhanced the soil-water retention capabilities of the gel, illustrating its potential as an efficient water-retaining agent. Similarly, El-Naggar et al. [65] explored the synthesis of carboxymethyl cellulose/acrylic acid polymers using e-beam radiation, focusing on polymers that exhibit pH and temperature sensitivity. Their research highlighted how the swelling behavior and responsiveness of the gel in water are intricately linked to the applied electron-beam radiation dose. Notably, gels irradiated at a dose of 50 kGy exhibited a markedly higher swelling rate in water compared to those subjected to 80 kGy. Furthermore, gels prepared at 80 kGy demonstrated pH responsiveness but lacked temperature sensitivity, whereas those irradiated at 50 kGy showed temperature responsiveness without pH sensitivity. This distinct behavior underscores the capability of e-beam radiation to modulate the properties of hydrogels for targeted applications.

The utilization of e-beam radiation in the synthesis of hydrogels exemplifies the method’s advantages in achieving precise control over material properties, thereby opening a new avenue for the design and development of advanced materials with targeted functionalities. These advancements are particularly relevant in fields requiring materials with specialized characteristics, such as biomedicine, agriculture, and environmental management, where the unique benefits of e-beam technology can be fully exploited.

#### 3.3.3. Ultraviolet Radiation

Ultraviolet (UV) radiation emerges as an exceptionally efficient, environmental-friendly, and cost-effective method for initiating polymerization processes. Its comparative advantages over traditional polymerization techniques include a streamlined process, reduced reaction times, ease of operation, and the ability to conduct reactions at ambient temperatures. These characteristics make UV radiation a compelling choice for synthesizing and modifying polymers, especially when considering the sustainability and efficiency of manufacturing processes.

Kumar et al. successfully harnessed UV radiation to synthesize a composite film made from starch and microcrystalline cellulose [66]. In their methodology, sodium benzoate was utilized as a photosensitizer, and glycerin served as a plasticizer. Their findings indicate that the tensile modulus and strength of the film show corresponding enhancement as the exposure and radiation time increase. This improvement underscores the effectiveness of UV radiation in facilitating the formation of robust polymer networks. Similarly, Attia et al. exploited UV radiation for the modification of cellulose, incorporating polypyrrole/silver to create nanocomposite membranes in a singular step [67]. The utilization of UV radiation in this context not only simplified the initiation process but also expedited the polymerization, highlighting the capability of this method to efficiently produce materials with enhanced properties.

The adoption of UV radiation for polymer synthesis and modification offers a pathway towards more sustainable material production. Its ability to induce polymerization under benign conditions, without the need for elevated temperatures or harsh chemicals, aligns with the growing emphasis on green chemistry principles. Moreover, the simplicity and efficiency of UV-initiated processes hold significant promise in the development of innovative materials with tailored properties, opening new avenues in materials science and engineering. These advancements are particularly pertinent in fields requiring advanced materials, such as biomedical engineering, packaging, and environmental sustainability, where the unique advantages of UV radiation can be fully leveraged.

#### 3.3.4. Microwave Radiation

Microwave radiation stands out as a highly efficient method in the field of polymer chemistry, particularly for its ability to significantly reduce reaction times, conserve energy, ensure cleanliness, and deeply penetrate polymer compounds, thereby improving product performance. These advantages have garnered the attention of researchers exploring polymerization reactions, favoring microwave radiation over conventional heating methods.

Sun et al. [68] exploited microwave radiation to synthesize a salt-resistant hydroxyethyl cellulose superabsorbent resin, demonstrating the method’s efficiency. Their study indicated that optimal conditions—400 W of microwave power, intermittent heating for 12 min, an AM/MAA molar ratio of 0.45, a 3.0% hydroxyethyl cellulose concentration, 2.0% crosslinking agent dosage, and 0.15% initiator dosage—yielded a superabsorbent resin with a maximum brine absorption rate of 167 g/g. Similarly, Mishra et al. [69] utilized microwave radiation to synthesize a carboxymethyl cellulose/acrylic acid polymer, which was subsequently applied as a flocculant for river purification. These examples underscore the potential of microwave radiation to enhance polymer synthesis processes, making them more efficient and environmentally friendly.

Cellulose, with its abundance of hydroxyl groups, offers excellent potential for crosslinking through hydrogen bonding with other materials, resulting in superior physical properties. Its inherent thermal stability, mechanical robustness, environmental compatibility, and broad electrochemical stability window make very attractive for various applications. Consequently, cellulose has been increasingly reported as an additive in hydrogel materials, where it significantly contributes to the composite material’s buffering effect against external stresses through non-covalent and recoverable physical interactions. This interaction confers strong flexibility and shear resistance at the macro level.

Moreover, cellulose-based hydrogels often exhibit porous structures with tunable pore sizes, enhancing ionic conductivity. This characteristic, coupled with reinforcing capabilities of cellulose, leads to improved hydrogel electrolytes in terms of structural stability, interface adhesion, and tensile properties. The multifaceted benefits of cellulose underscore its value as a reinforcing agent in hydrogel components, highlighting its role in promoting the development of high-performance flexible devices.

## 4. Applications of Cellulose Hydrogels in ZIBs

Despite significant efforts to develop flexible ZIBs, challenges remain in meeting the diverse requirements of practical applications, such as flexibility, wearability, comfort, durability, safety, and wettability. A potential solution to these challenges is developing new functional hydrogel electrolytes that incorporate cellulose hydrogel composites. However, the design and integration of cellulose hydrogels into flexible batteries must be carefully executed. This involves enhancing the ionic conductivity, mechanical properties, and responsiveness to environmental changes. Recently, researchers also tried to introduce smart features that allow the batteries to operate efficiently within varied challenging conditions.

While flexible ZIBs exhibit promising performance in laboratory settings, their transition to real-world applications encounters significant hurdles, particularly due to the limitations of hydrogel electrolytes under extreme environmental conditions. The intrinsic property of hydrogels to retain water, granting them liquid-like characteristics, poses a challenge at temperatures below freezing point (0 °C) and in high-temperature environments (e.g., 50 °C), as well as under physical deformation.

At low temperatures, the freezing of water within hydrogels disrupts its internal network structure, leading to a loss of elasticity and flexibility. This frozen state eliminates the hydrogel’s ability to adapt its shape, which is critical for maintaining effective contact in battery systems. Moreover, the solubility of water decreases significantly in cold conditions, causing salt precipitation within the hydrogel and a consequent reduction in ionic conductivity—an essential factor for the performance of electrolyte. Conversely, at elevated temperatures, the hydrogel faces accelerated water evaporation, which not only impacts its structural integrity through shrinkage and deformation but also exacerbates the loss of ionic conductivity due to salt precipitation. This dehydration effect can severely impair the properties of hydrogel, leading to increased interfacial resistance when integrated into a ZIB. Such resistance is detrimental to battery efficiency, as it hinders the smooth flow of ions in the electrolyte between the electrodes.

Addressing these temperature-related challenges requires innovative approaches to hydrogel electrolyte design. Strategies might include the development of hydrogels with lower freezing points, enhanced thermal stability, or the incorporation of additives that can retain water or improve ionic conductivity under varying temperature conditions. By overcoming these obstacles, flexible ZIBs could achieve the robustness needed for practical applications, expanding their utility in a wide range of environmental conditions.

### 4.1. Anti-Freezing ZIBs

**Figure 5:**
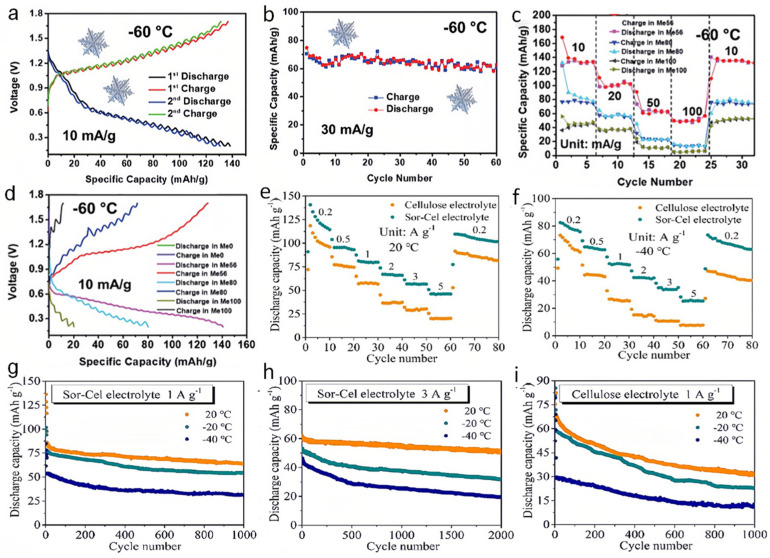
(**a**,**b**) Charge–discharge profiles and (**c**) rate performance of ZIBs with methanol molar ratio of 56% at −60 °C. (**d**) Charge–discharge profiles of ZIBs with different methanol molar ratio at 10 mA g^−1^ at −60 °C. Rate performance of ZIBs with different hydrogel electrolytes (**e**) at −20 °C and (**f**) at −40 °C. Cycling performances at 20, −20, and −40 °C of Zn–PANI batteries with (**g**) Sor-Cel electrolyte at 1 A g^−1^, (**h**) Sor-Cel electrolyte at 3 A g^−1^, and (**i**) cellulose electrolyte at 1 A g^−1^. Reproduced with permission from Ref. [72]. Copyright© The Royal Society of Chemistry, 2021.

For flexible ZIBs with hydrogels as electrolytes, the critical challenges are preventing dehydration at high temperatures, freezing at low temperatures, and thermal shock, which severely restrict the long-term reversibility. To make ZIBs work well under the aforementioned harsh conditions, functional hydrogels with anti-freezing, anti-dehydration, and thermo-response have been explored for temperature-adaptive ZIBs [70,71]. Xu et al. introduced an innovative approach for enhancing the performance of flexible Zn-based batteries under extreme cold conditions [72]. They developed an anti-freezing hydrogel by integrating cellulose nanofibers (CNFs) with PAM in a methanol/water hybrid solvent, aimed at a Zn-PANI (polyaniline) battery system. The utilization of methanol as part of the solvent composition reduces the freezing point of hydrogel electrolyte effectively, thereby maintaining its superior mechanical properties, such as flexibility and resilience to repeated bending and twisting, even at the low temperatures of −20 °C. A remarkable aspect of this development is that the flexible Zn-MnO_2_ battery works efficiently at significantly low temperatures, with an unprecedented capacity (Figure 5a,c) and cycling performance at −60 °C (Figure 5b,d). This achievement is attributed to the substitution of methanol for free water within the hydrogel, which lowers its freezing point and prevents the loss of electrolyte functionality in cold environments.

Based on this concept, sorbitol, a compound characterized by its rich -OH groups and long molecular chain, was later used to further modify the cellulose hydrogels for the Zn-PANI battery [73]. The modified hydrogel showcase not only enhanced mechanical properties and improved adhesion but also had an ultralow freezing point of −40 °C (Figure 5e,f). As a result, the flexible Zn-PANI battery equipped with this modified cellulose hydrogel as an electrolyte exhibits commendable electrochemical performance at cold conditions (Figure 5g–i). These advancements underscore the potential of using hybrid solvents and specific additives to overcome the challenges posed by low-temperature environments to the functionality of flexible ZIBs. By designing the composition of hydrogel electrolytes, researchers can significantly extend the operational range of these batteries, making them more suitable for a wider array of applications, particularly in regions subjected to cold climate.

Ionic liquids (ILs) have garnered significant attention in the field of flexible ZIBs, thanks to their unique properties, including low volatility, good chemical stability, high temperature resistance, and impressive ionic conductivity [74]. Their ability to remain liquid and conductive without evaporating, much like water, yet resist the formation of ice crystals under freezing conditions, makes them ideal for improving the performance of hydrogel electrolytes in extreme environments. The mechanism by which ionic liquids prevent ice formation involves a decrease in the chemical potential of solution, attributable to the entropy effect. ILs containing ionized molecules similar to H_2_O molecules reduce the presence of “free water” that would otherwise form ice crystals. When mixed with water, the anions and cations of ILs disrupt the network structure that water molecules need to form ice, thus significantly hindering ice crystallization.

Wang et al. [75] capitalized on these properties of ILs to develop cellulose antifreeze hydrogel electrolytes. They dissolved cellulose in benzyl trimethylammonium hydroxide (BzMe_3_NOH) and chemically crosslinked it with epichlorohydrin (ECH). The resulting hydrogel electrolyte showcased remarkable characteristics: over 90% transparency at −24 °C, an ionic conductivity of 2.37 × 10^−2^ S∙cm^−1^, and an elongation at a break of 219% at −10 °C. In stark contrast, hydrogel electrolytes without ionic liquids rapidly froze at −40 °C, became turbid due to ice crystal formation, and failed to regain their original form post-bending.

**Figure 6:**
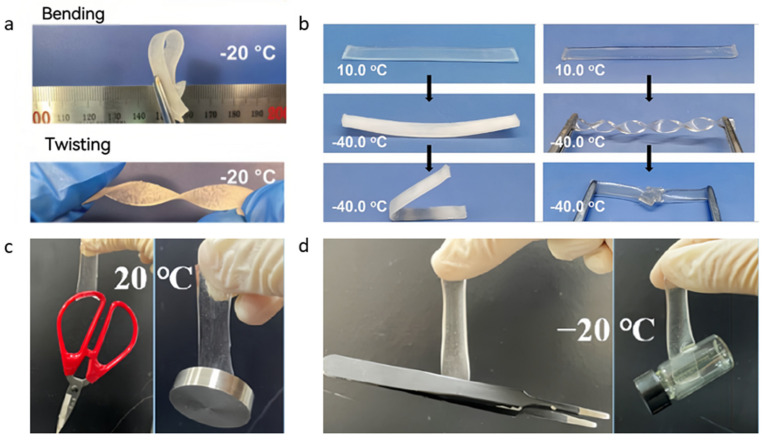
Mechanical performance of the MgVO/Zn (ssBs) battery based on the CNF–PAM hydrogel electrolyte (**a**) bending and (**b**) twisting after freezing at −20 °C for two days. (**b**) Photographs of cellulose hydrogel with pure water as solvent (CH) (left panels) or with CCH (right panels) at 10 and −40 °C. Adhesion evaluation of Sor-Cel electrolyte at (**c**) 20 °C and (**d**) −20 °C. Reproduced with permission from Ref. [75]. Copyright© The Royal Society of Chemistry, 2020.

Conversely, the hydrogel electrolytes containing BzMe_3_NOH maintained their hydrogel-like properties even at −40 °C, retaining transparency, stretchability, and elasticity without any visible damage when bent or knotted (Figure 6a,b). This study demonstrates the potential of ILs to endow flexible ZIBs with frost resistance, thus expanding their usability in cold environments. This innovation not only broadens the operational temperature range of ZIBs but also enhances their durability and reliability, rendering them more suitable for practical applications where extreme temperatures are a concern. Recently, antifreeze PAM hydrogel electrolytes have been designed by using zwitterionic polyionic liquids (PILs), which contain equal positively and negatively charged groups in a unit. The resulting amphoteric PIL hydrogel electrolyte achieved a 900% super stretch even at −20 °C (Figure 6c,d).

**Figure 7:**
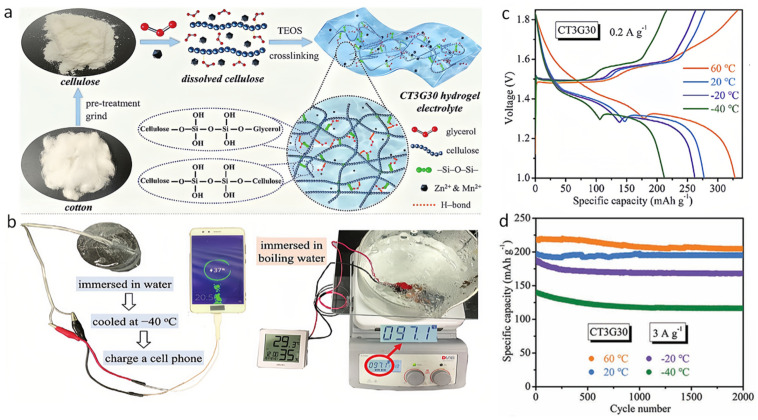
(**a**) Scheme of a cellulose-integrated hydrogel electrolyte. (**b**) Demonstration of charging a cell phone with three tandem Zn–MnO_2_ batteries with CT3G30, and demonstration of one Zn-MnO_2_ battery with CT3G30 immersed in boiling water to power an electronic clock. (**c**) Charge–discharge curves at 0.2 A g^−1^, and (**d**) cycling performance of the Zn–MnO_2_ batteries with CT3G30 at different temperatures. Reproduced with permission from Ref. [76]. Copyright© Wiley, 2021.

Recently, Chen et al. reported the design of hydrogels for flexible ZIBs by creating a functional integrated hydrogel electrolyte by employing cellulose as the substrate, glycerol as an antifreeze component, and tetraethyl orthosilicate (TEOS) as a crosslinking agent (Figure 7a) [76]. This composition allows TEOS to covalently bond with glycerol and cellulose, forming a stable three-dimensional network. Additionally, hydroxyl groups on the cellulose chains engage in reversible hydrogen bonding, which interacts with water molecules to reduce their activity. This optimized hydrogel electrolyte exhibits a suite of exceptional characteristics: high ionic conductivity, remarkable mechanical strength (with a tensile strength increase of 846.5%), an ultra-low freezing point of −64.6 °C, impressive self-healing capabilities (self-healing rate of 82.6%), satisfactory adhesion, and robust heat resistance up to 100 °C. Notably, its porous structure and antifreeze properties enable it to achieve a high ionic conductivity (19.4 mS∙cm^−1^) at 40 °C, surpassing previous hydrogel electrolytes designed for ZIB applications. Remarkably, the hydrogel electrolyte maintains functionality under severe deformation at −40 °C, even when sealed in an ice cube or immersed in boiling water (Figure 7b).

Furthermore, Zn-MnO_2_ batteries equipped with this type of synthetic hydrogel as the electrolytes demonstrate an excellent specific capacity of 211.8 mAh·g^−1^ at −40 °C (Figure 7c) and an enduring cycle life of 2000 cycles between 60 °C and −40 °C (Figure 7d). Beyond its exceptional performance in extreme conditions, this hydrogel electrolyte effectively mitigates the formation of Zn dendrites and minimizes side reactions, addressing common challenges in ZIBs. The breakthroughs achieved by Chen et al. [76] underscore the potential of meticulously engineered hydrogel electrolytes in revolutionizing ZIBs, particularly for applications requiring operation across a wide range of temperatures and under physically demanding conditions. This advancement paves a guideline for the development of more resilient and efficient energy storage solutions, broadening the scope of applications for ZIBs in modern technology and devices.

### 4.2. Self-Healing ZIBs

In addition to anti-freezing function, the concept of self-healing hydrogels draws inspiration from the natural, efficient self-repair processes observed in plant and animal tissues. These advanced materials are engineered to autonomously mend themselves after damage, mirroring biological healing. Flexible ZIBs are similarly expected to possess such self-recovery capability by employing self-healing hydrogels as electrolyte [77,78,79,80]. A notable development comes from the Jiang team that a new type of organic hydrogel was crafted [81]. This hydrogel, made through a sol–gel conversion of PVA and CNFs in a dimethyl sulfoxide (DMSO)/water system, demonstrates exceptional characteristics: high elasticity (up to 660%), strength (up to 2.1 MPa), energy absorption (5.25 MJ m^−3^), and transparency (up to 90%). It maintains flexibility and conductivity (1.1 S∙m^−1^) even at −70 °C. Achieved through simple sol–gel conversion followed by immersion in a salt solution, this organic hydrogel showcases the synergistic effects of CNFs in boosting both mechanical properties and ionic conductivity. These developments underscore the potential of cellulose-based hydrogels in creating high-performance, environmentally adaptable ZIBs, pushing forward the boundaries of energy storage technology.

The self-healing mechanisms in hydrogels are primarily categorized based on the nature of the dynamic bonds involved: dynamic non-covalent bonds and dynamic reversible covalent bonds [82]. Dynamic non-covalent bond mechanisms in self-healing cellulose hydrogels encompass a variety of interactions, including hydrogen bonds, hydrophobic interactions, host–guest interactions, ionic bonds, and electrostatic actions. These mechanisms rely on the inherent ability of the molecular components of hydrogels to re-establish bonds after being disrupted, facilitating the self-healing process without the need for external stimuli. A notable example of this technology is presented by Wang et al. [29], who developed a self-healing cellulose hydrogel utilizing UPy (ureido-pyrimidinone) multiple hydrogen bonding. In their approach, UPy groups were grafted onto the surface of CNCs (CNC-UPy), which were then integrated with PVA to form a PVA/CNC-UPy supramolecular hydrogel. This innovative hydrogel demonstrated the capability to self-repair within just 3 min at room temperature, showcasing the potential of dynamic non-covalent bonding mechanisms in creating robust, self-healing materials.

The dynamic reversible covalent bond mechanism in self-healing cellulose hydrogels incorporates reactions such as borate, imide, disulfide, hydrazone, and Diels–Alder, providing these materials with enhanced self-healing stability through stronger molecular interactions. This bonding mechanism offers greater durability compared to dynamic non-covalent bonds, making it particularly suitable for applications requiring robust self-repair capabilities. Huang et al. utilized a mechanical ball milling “one-pot method” to produce PVA/microfibrillated self-healing cellulose hydrogels based on dynamic borate ester bonds [83]. Their research highlighted that incorporating microfibrillated cellulose significantly bolstered both the mechanical properties and the self-healing efficiency of hydrogels, enabling them to self-heal within 10 min after being cut and re-joined.

**Figure 8:**
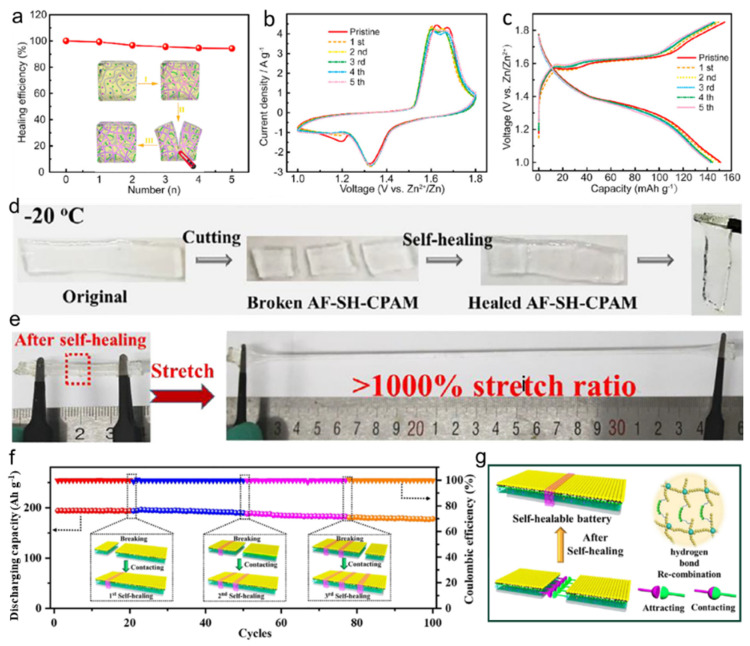
(**a**) Healing efficiency of a self-healable hydrogel as electrolyte and (**b**) CV curves and (**c**) GCD profiles of the flexible ZIBs before and after multiple cutting/self-healing cycles. Reproduced with permission from Ref. [84]. Copyright© Elsevier, 2021. (**d**) The self-healing process under −20 °C of the AF-SH-CPAM. (**e**) The AF-SH-CPAM after self-healing showing a high stretching property of over 1000%. (**f**) Cycle performance at 0.5 A g^−1^ of the ZIB with AF-SH-CPAM as electrolyte at original state and after different cutting/self-healing times. (**g**) Schematic diagram of self-healing mechanism of the ZIB with AF-SH-CPAM. Reproduced with permission from Ref. [85]. Copyright© Elsevier, 2022.

Recently, Ling et al. synthesized a self-healable hydrogel electrolyte with rigid-flexible backbones by crosslinking polymerization and composite strengthening [84]. Owing to the porous crosslinking network and more hydrophilic groups, the self-healable hydrogel possesses a high ionic conductivity of 23.1 mS cm^−1^ at 25 °C and excellent mechanical strength. When used in the ZIBs, the hydrogel possessed a high healing efficiency of above 95% (Figure 8a) after five cutting/self-healing cycles. The good repeatable CV curves of the flexible ZIB suggested that the self-healing process has little influence on the electrochemical polarization (Figure 8b). Moreover, during the five cutting/self-healing cycles, the flexible ZIB still maintained uniform GCD curves at 3.0 A g^−1^ (Figure 8b). Qu’s group reports an anti-freezing and self-healing crosslinked polyacrylamide polyelectrolyte (AF-SH-CPAM) prepared by in-situ polymerization [85]. At −20 °C, AF-SH-CPAM still displays an excellent self-healable feature (Figure 8d). Impressively, after the first cutting/self-healing cycle, the stretch ratio of recovered AF-SH-CPAM still remains well above 1000% (Figure 8e). As shown in Figure 8f, before cutting, the ZIB with AF-SH-CPAM as the electrolyte shows a high specific capacity of 194 mAh g^−1^ and a coulombic efficiency of 100% for 20 cycles at 0.5 A g^−1^. After cutting in halves and subsequent self-healing for half an hour, the self-healed ZIB delivers a capacity of 193.7 mAh g^−1^ with a coulombic efficiency of 99% and maintains a high capacity of 189.4 mAh g^−1^ at the 50th cycle. Impressively, the ZIB still retains a specific capacity of 188.2 mAh g^−1^ and 180.5 mAh g^−1^ after the seconnd and third cutting/self-healing cycle, respectively. After 100 g cycles, the self-healed ZIB with three cutting/self-healing processes can stably provide a specific capacity of 177.8 mAh g^−1^, about 91.6% of the original capacity. The good self-healable ability can be attributed to molecular interaction of copious hydroxyl and carbonyl groups on the surface of AF-SH-CPAM around the fracture, which recovers the mechanical property and ion transmissions of hydrogel electrolyte.

The development of self-healing cellulose hydrogels represents a significant advancement in material science, offering promising applications in areas where durability and longevity are critical. By mimicking natural healing processes, these hydrogels pave the way for the next generation of smart materials that can significantly reduce maintenance needs and extend the lifespan of products and structures.

### 4.3. Smart ZIBs

Obviously, extreme environments will ultimately destroy the performance of flexible ZIBs with hydrogel, because the hydrogel limits the range of operating conditions. On the other hand, heat accumulates rapidly during the ultrafast charging and discharging of batteries or under extreme conditions, such as an internal short circuit, which lead to severe thermal shock or even explosion [86,87]. Moreover, overcharge or overdischarge will inevitably degrade the electrochemical performance, resulting in poor rated capacity or cycling stability. It could become worse when overcharge or overdischarge triggers the decomposition reaction of water in aqueous electrolyte accompanying the H_2_ and O^2^ evolution. This would increase the pressure inside the working ZIBs and induce the explosion to some extent in the limited space. Flexible ZIBs with hydrogels as electrolyte are praised for high safety due to the inherent water quality and delicate design of hydrogel.

**Figure 9:**
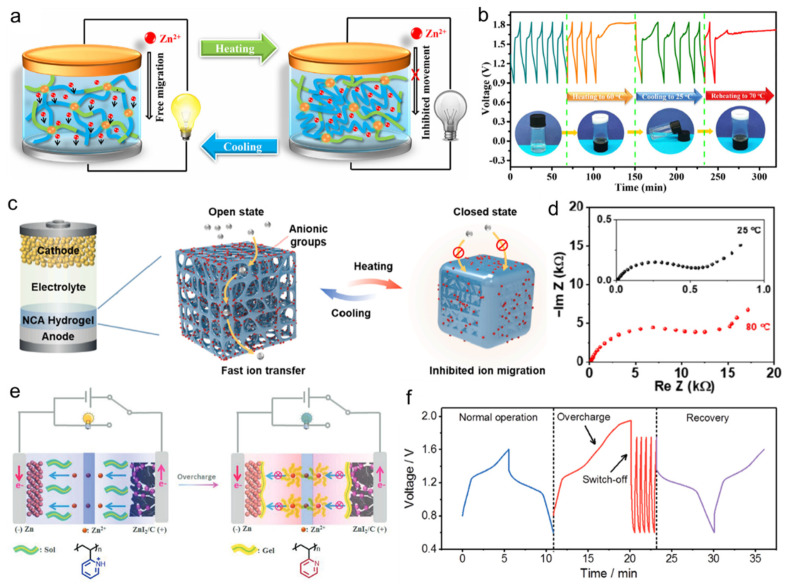
(**a**) Illustration of a thermoresponsive sol–gel transition system in batteries and (**b**) charge–discharge cycles of the ZIB with sol–gel electrolyte at different temperatures. Reproduced with permission from Ref. [89]. Copyright© Elsevier, 2018. (**c**) Intrinsic thermal self-regulating feature in battery operation by the hydrogel film and (**d**) impedance spectra at different temperatures. Reproduced with permission from Ref. [90]. Copyright© Elsevier, 2024. (**e**) Illustration of the self-protection function of ZIBs with a pH-responsive electrolyte at the overcharge state, and (**f**) galvanostatic charge/discharge curves of ZIBs in the voltage window of 0.6–1.6 V before and after the overcharge to 1.95 V. Reproduced with permission from Ref. [91]. Copyright© Wiley, 2020.

Smart responsive hydrogel electrolyte can be incorporated into flexible ZIBs to prevent thermal runway and overcharge/discharge, endowing the ZIBs with a smart response [88]. Zhi’s group reported a smart thermoresponsive electrolyte that can prevent thermal runaway via a fast and reversible sol–gel transition [89]. When assembled into a rechargeable Zn/a-MnO_2_ battery, zinc ions can freely migrate between cathode and anode because the sol–gel electrolyte is in the sol state at low temperatures. Once heated to a certain temperature, the sol–gel electrolyte forms a stationary hydrogel that inhibits the zinc-ion diffusion and shuts down the batteries (Figure 9a). Importantly, the transition is reversable, and electrochemical performance can be recovered after cooling down. As shown in Figure 9b, the thermoresponsive batteries with PNA sol–gel electrolyte initially showed good capacity retention over five cycles at 25 °C. When heating to 70 °C, the increasing voltage cannot reach 1.8 V over a long charging time. After the temperature goes down, the battery resumes normal charging process. Yang and coworkers propose a thermoresponsive polyanionic hydrogel film as a bifunctional layer in ZIBs (Figure 9c), where the thermoresponsive hydrogel shrinks at the high temperature within several seconds, impeding zinc-ion transport and leading to a self-protective state [90]. As shown in Figure 9d, at the high temperature of 80 °C, the internal charge-transfer resistance of the ZIB demonstrates more than 20-fold increase, resulting in sluggish kinetics and triggering self-protection, which effectively alleviates battery thermal runaway.

In addition to thermal self-protection, overcharge self-protection is also very important for the safety of batteries. To achieve this goal, a smart pH-responsive electrolyte was designed by Feng’s group to assemble in a stimulus-responsive ZIB (Figure 9e) [91]. The pH increase in the overcharged ZIBs induces the fast transition of poly(2-vinylpyridine) (P2VP) from a hydrophilic soluble state to a hydrophobic gel state in less than 30 s. The de-protonation of quaternary pyridinic groups in electrolyte leads to the internal resistance increase in the overcharged ZIBs by four orders of magnitude. As demonstrated in Figure 9f, once ZIBs with P2VP electrolyte were overcharged at 1.6 A g^−1^ to 1.95 V, the subsequent discharge time dramatically decreases, indicating an obvious switch-off behavior of ZIBs controlled by pH value at the overcharge state. This work provides a new avenue for developing smart aqueous Zn batteries with self-protection ability at the overcharge state.

## 5. Summary and Outlook

This review discusses recent advances of the hydrogel electrolyte in flexible ZIBs, especially focusing on the unique benefits offered by cellulose modification. These hydrogels combine the roles of electrolytes and membranes, allowing for fast ion transport without traditional membranes and contributing to the good flexibility of ZIBs. The design of these ZIBs prioritizes the softness and ionic conductivity of hydrogel. Research is expanding into making ZIBs more versatile, exploring properties like extreme durability, high stretchability, temperature resistance, and self-healing capabilities. These advancements aim to enhance battery performance and broaden their application in various fields.

The realm of functional cellulose hydrogels is witnessing remarkable achievements. Conductive cellulose hydrogels, which incorporate electrolyte salts into a cellulose hydrogel network, emerge as functional soft materials with inherent conductivity [92,93]. These hydrogels can be easily prepared and exhibit high mechanical strength, excellent conductivity, and robust frost resistance. Cellulose serves as the primary scaffold in these hydrogels, offering a three-dimensional support network and an abundant pore structure for ion-conducting polyelectrolytes [94,95,96].

The exploration of functional cellulose hydrogel electrolytes in flexible ZIBs presents a significant opportunity for innovation in the field. By adapting and enhancing these functional methods, it is possible to create novel cellulose hydrogel electrolytes specifically designed for flexible ZIBs. These hydrogels can then be evaluated for their electrochemical behavior and functional performance, potentially leading to advancements in battery technology. However, the scalability of flexible ZIBs poses a substantial challenge. The scale up of these batteries is hindered by several issues, particularly the irreversible side reactions associated with Zn dendrite formation [97], hydrogen evolution reaction, corrosion, and surface passivation. These issues can severely impact the stability of Zn anode, compromising the overall performance and longevity of ZIBs.

Cellulose hydrogel electrolytes, with unique quasi-solid-state characteristics, offer a promising approach to mitigating the HER in mild electrolyte. Nevertheless, their capacity to suppress the formation of Zn dendrite is often limited by their relatively poor mechanical strength. Addressing this limitation requires further research to enhance the mechanical properties of cellulose hydrogel electrolytes without compromising their beneficial characteristics. By achieving this balance, it may be possible to significantly improve the stability and scalability of flexible ZIBs, making them more viable for various applications.

Self-healing cellulose hydrogels, thanks to their dynamic reversible crosslinking, offer numerous advantages over traditional cellulose hydrogels, such as extended material lifespan and broader application potential. However, a main challenge in this field is achieving a balance between self-healing ability and mechanical properties, as these two factors tend to be inversely related. Future research will likely focus on refining the construction strategies and incorporating multiple reversible crosslinked networks to overcome this limitation, paving the way for more resilient and versatile self-healing materials. Despite obvious progress on functional hydrogels, smart ZIBs with targeted functions are still in their infancy and far from industrial production. It is imperative to deepen the understanding on ion transport in hydrogels and the interaction between water molecules and hydrogels. With no doubt, hydrogels are the key material for flexible ZIBs with a market niche in widespread wearable electronics.

## Figures and Tables

**Table 1:** Representative hydrogels used for electrolytes including their molecular structures, general characteristics, and ionic conductivity for ZIBs [25].

Names of Hydrogel	Molecular Structures	Functional Groups	Features	Ionic Conductivity (S∙cm^−1^)	Cathode Material	Mechanical Properties
Poly(ethylene oxide) (PEO)	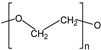	Hydroxyl	Reusable	1.09 × 10^−6^– 6.33 × 10^−3^	MnO_2_ 140 mAhg^−1^ 0.5 mAcm^−2^	Physical crosslinking, flexible
Polyacrylamide (PAM)	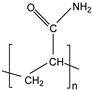	Amide	Reusable	2.15 × 10^−3^	V_2_O_5_ 271 mAhg^−1^ 2C	Chemical crosslinking, stretchable
Polyacrylic acid (PAA)	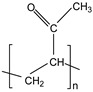	Carboxyl	Self-healable	0.288	MnO_2_ 5.6 mAhcm^−2^ 0.5 mAcm^−2^	Physical/ chemical crosslinking, stretchable
Sodium polyacrylate (PANa)	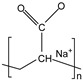	Sodium carboxylate	Self-healable, alkali resistant	0.17	NiCo 259 mAhg^−1^ 5.8 C	Physical crosslinking, stretchable

**Table 2:** Cellulose derivatives and the corresponding reaction mechanisms [34].

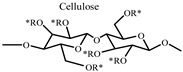	
Cellulose derivatives and R*	Reaction mechanism
MC [R*: H, CH_3_]	RONa + CH_3_Cl→ROCH_3_ + NaCl
HPC [R*: H, CH_2_CH(OH)CH_3_]	RONa + CH_2_CH(OH)CH_3_Cl→ROCH_2_CH(OH)CH_3_ + NaCl
HPMC [R*: H, CH_2_CH(OH)CH_3_]	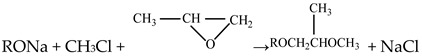
CMCNa [R*: H, CH_2_COONa]	RONa + ClCH_2_COONa→ROCH_2_COONa + NaCl
HEC [R*: H, CH_2_CH_2_OH]	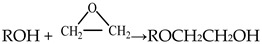
EC [R*: H, CH_2_CH_3_]	RONa + CH_2_CH_3_Cl→ROCH_2_CH_3_ + NaCl

MC: methyl cellulose; HPC: hydroxypropyl cellulose; HPMC: hydroxypropyl methylcellulose; CMCNa: carboxymethyl cellulose sodium salt; HEC: hydroxyethyl cellulose; EC: ethyl cellulose.
